# Biotinylated Chlorin and Its Zinc and Indium Complexes: Synthesis and In Vitro Biological Evaluation for Photodynamic Therapy

**DOI:** 10.3390/ph10020041

**Published:** 2017-04-15

**Authors:** Meden F. Isaac-Lam, Dewana M. Hammonds

**Affiliations:** Department of Chemistry and Physics, Purdue University Northwest, 1401 S US Hwy 421, Westville, IN 46391, USA; dhammon@pnw.edu

**Keywords:** photodynamic therapy, PDT, chlorin-biotin conjugate, zinc chlorin-biotin complex, indium chlorin-biotin complex, vitamin-targeted cancer therapeutics

## Abstract

The synthesis and characterization of biotinylated chlorin photosensitizer and the corresponding zinc and indium complexes are described for potential applications in photodynamic therapy (PDT) for cancer. Phototoxicity of the biotin-chlorin conjugate and the metallated complexes was determined in colon carcinoma CT26 cell lines known to overexpress biotin (Vit B_7_) receptors. Cell survival assay indicated that the biotinylated chlorin and indium complex showed increased cell growth inhibition than the zinc complex and the starting chlorin (methyl pheophorbide). Fluorescence microcopy studies revealed the generation of apoptotic cells upon light irradiation of colon cells treated with the indium complex. Targeting biotin receptors in cancer cells can improve specificity of photosensitizers for PDT applications.

## 1. Introduction

Photodynamic therapy (PDT) is a cancer treatment that involves delivery of a fluorophore known as photosensitizer (PS) to tumor tissues upon systemic administration and activation with visible light (600–800 nm) in the presence of endogenous oxygen [1]. Excitation of PS with light in the red or near infrared (NIR) region generates cytotoxic reactive oxygen species (ROS), including singlet oxygen (^1^O_2_) to cause irreversible destruction of tumor cells, and to induce immune inflammatory responses and damage to tumor microvasculature. PDT is an FDA-approved treatment for bronchial, esophageal, gastric, cervical, skin, head, and neck cancers. However, PDT has certain limitations. Photosensitizer selectivity and efficacy which continue to be the major barrier for widespread acceptance of PDT in clinical practice must be improved to optimize anticancer treatment [2,3,4,5]. Characteristics of ideal photosensitizers include low dark toxicity, high selectivity for tumors over normal tissues, high quantum yield of light-induced triplet state oxygen formation, and rapid clearance from the body [6,7,8,9]. In PDT and in conventional cancer chemotherapy, increasing the amount of anticancer drugs is often necessary to achieve a linear response in killing cancer cells causing undesirable increase in toxicity to normal healthy cells due to non-selective tissue targeting.

Targeted therapy for cancer treatment exploiting ligands for selective delivery of pharmaceuticals to malignant cells is a strategy to improve anticancer drugs for treatment and imaging [10,11]. Cancer cells overexpress tumor-specific receptors which can serve as targets to deliver photosensitizers into tumors. In particular, rapidly proliferating cancer cells require certain vitamins to sustain growth-causing vitamin receptors to be overexpressed on the cancer cell surface. B vitamins such as folic acid (B_9_), biotin (B_7_), riboflavin (B_2_), and cobalamin (B_12_) are essential vitamins for survival of all living cells, including the growth of cancerous cells. In contrast to folate receptors [12,13,14] which gained considerable attention as excellent biomarkers for targeted drug delivery, there are only few studies done on biotin receptors. Biotin is a water-soluble micronutrient that plays an essential role in the growth, development, differentiation, and normal function of animal cells. It serves as a cofactor responsible for several carboxylase enzymes involved in key biosynthetic metabolic processes of fats and amino acids and in gluconeogenesis. It has been shown that biotin receptors are overexpressed more than folate or vitamin B_12_ receptors in several cancer lines such as colon, breast, renal, lung, and leukemia cell lines [15,16,17]. Targeted delivery of cytotoxic agents using biotin as a cancer-specific ligand provide an attractive alternative to increase selectivity for cancer therapy. Previous studies have used biotinylated conjugates including ferrocene [18], paclitaxel [19], coumarin [20], and dendrimers [21,22] as carriers to enhance cancer cell-specific uptake.

Several photosensitizers derived from hematoporphyrin have been developed for PDT treatment of melanoma, cancer of the lung, intestinal tract, and Barrett’s esophagus [6,7,8]. Chlorins or chlorophyll-*a* derivatives have been shown to exhibit low dark toxicity and to generate singlet oxygen species upon light irradiation [7,8,9,23,24,25]. In the present study, chlorin derivative conjugated at 13^1^ position was synthesized including its zinc(II) and indium(III) metal complexes to assess the photodynamic effect of conjugation of the photosensitizer with biotin, and to evaluate the cytotoxicity of the conjugate against cells that are known to overexpress biotin receptors. We describe herein the synthesis of new chlorin derivatives conjugated to vitamin B_7_ that will target biotin receptors overexpressed in colon cancer cells. This research, while consisting of only the synthesis of the new molecules and in vitro testing into cells, holds limitless opportunities for the further advancement of cancer research and treatment.

## 2. Results and Discussion

### 2.1. Chemistry

**Scheme 1:**
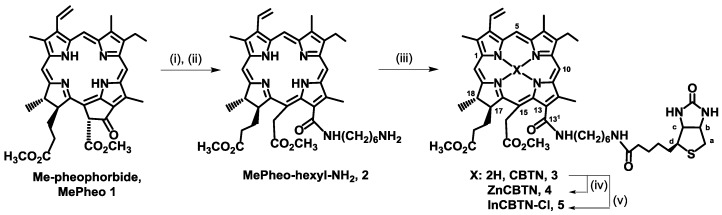
Synthesis of biotin-linked chlorin derivatives. Reaction conditions: (i) H_2_N(CH_2_)_6_NHBoc, CH_2_Cl_2_; (ii) trifluoroacetic acid (TFA); (iii) biotin, DMTMM; (iv) Zn(OAc)_2_ in MeOH; and, (v) InCl_3_ in MeOH.

**Figure 1:**
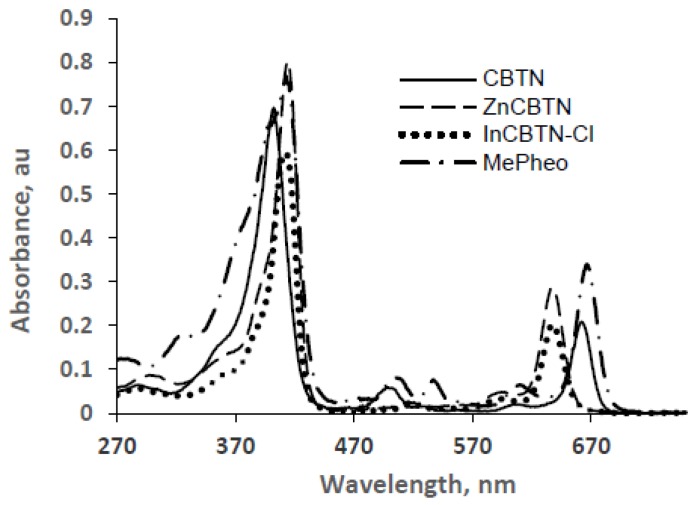
UV-vis absorption spectra of MePheo **1**, CBTN **3**, ZnCBTN **4**, and InCBTN-Cl **5** in dichloromethane.

Methyl pheophorbide *a* (**1**), a commercially-available starting material, was conjugated to biotin using a hexyl diamine linker to produce the target biotinylated chlorin derivative (**3**) in fair yield, using published peptide coupling procedure [26,27,28,29] as shown in Scheme 1. The target compound was converted into its zinc **4** and indium **5** complexes following published metalation protocols. Compounds **3**–**5** were purified by preparative silica gel TLC plate and the molecular structures were characterized by ^1^H-, ^13^C-, COSY, HSQC NMR, and MS spectral data. The presence of biotin in the conjugate is indicated by two sets of broad multiplets centered at 3.13 and 2.93 ppm and 3.06 and 2.96 ppm for chlorin-biotin **3** and its zinc complex **4**, respectively, and assigned to the two diastereotopic protons adjacent to the sulfur atom in the biotin ring. However, these two inequivalent protons collapsed into broad peaks in the indium complex overlapping with the other peaks in the chlorin macrocyclic ring. Figure 1 shows the UV-vis absorption spectra of the starting methyl pheophorbide **1**, and the target compounds CBTN **3**, and the corresponding zinc **4** and indium **5** complexes. Approximately 12 nm shift to longer wavelength of the Soret band and 25 nm shift to shorter wavelength are observed for zinc and indium complexes compared to unmetallated CBTN. The bathochromic (red, longer) and hypsochromic (blue, shorter) shifts for the Soret and the fourth Q band, respectively, are typically observed for metallated chlorin and porphyrinic compounds [30,31].

Conjugation of the photosensitizer to the biotin involved the attachment of a six-carbon spacer arm, which provides sufficient length between macrocyclic chlorin ring and the bicyclic biotin rings that would result in better binding potential to the biotin receptors expressed over the cancer cell surface. The extended length decreases the steric interaction caused by the chlorin macrocycle upon connecting with the biotin moiety. Attachment of a linker bridging a fluorophore to a vitamin folic acid was shown by other groups to enhance aqueous solubility and selectivity when tested in folate-receptor enriched human nasopharyngeal epidermoid carcinoma KB cells [32]. Previous studies also demonstrated that zinc [30,33,34,35,36] and indium [31,37,38] metal coordination to chlorins enhances in vitro oxidative cell damage and higher singlet oxygen production, respectively.

### 2.2. In Vitro Cellular Assay

Biotin as well as other vitamins are internalized by receptor mediated endocytosis and, specifically, biotin is taken up by the cells via a sodium dependent multivitamin transport system (SMVT) [15,16,17]. Chlorin photosensitizers are known to exhibit preferential localization in cancerous tissues [39] due to specific characteristics of cancer cells particularly decreased pH [40], increased permeability of tumor vasculature [41], overexpression of low-density lipoprotein (LDL; apoB/E) receptors [42], and presence of heme carrier protein [43]. PS can also accumulate in mitochondria via the peripheral benzodiazepine receptors located in the inner mitochondrial membrane [44]. It is likely that the same mechanistic pathways will come into play for the synthesized biotinylated chlorin derivative in this study with respect to its transport into cancer cells. Thus, based on the biotin-mediated transport system and chlorin macrocyclic ring serving as ligands for upregulated cancer cell surface receptors, linking biotin and a chlorin derivative will provide better photosensitizing activity for the PS than the nonconjugated entity.

**Figure 2:**
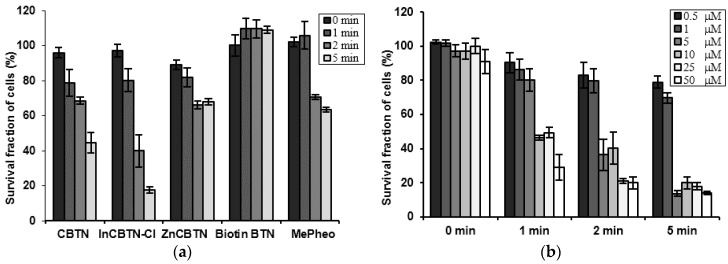
Bar plots of cell survival assay of colon cancer cell line. (**a**) After incubation with 5 μM photosensitizers (MePheo **1**, CBTN **3**, ZnCBTN **4**, and InCBTN-Cl **5**) and biotin, and irradiation for 0, 1, 2, and 5 min. (**b**) After incubation with varying concentrations of the indium (III) complex **5** and light irradiation for 0, 1, 2, and 5 min.

**Figure 3:**
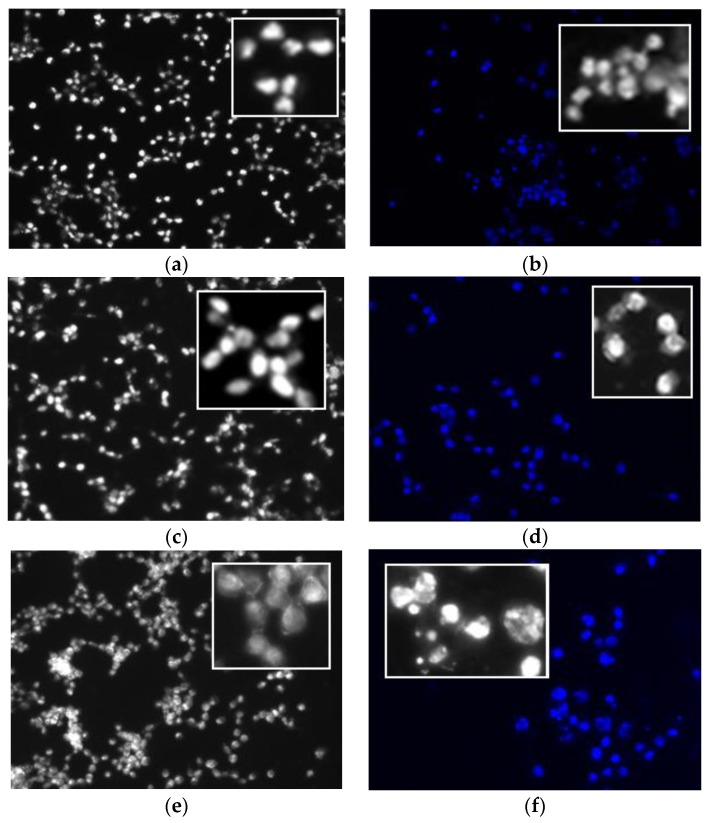
Fluorescence microscopy images of fixed human colon carcinoma cells. Grayscale and blue fluorescence images of CT cells alone stained with Hoechst 33258. Cell morphology of (**a**) untreated unirradiated cells (sham control); then, after treatment with 5 μM of (**b**) CBTN and irradiated for 2 min, (**c**,**d**) ZnCBTN dark and 2 min irradiation; and (**e**,**f**) InCBTN-Cl dark and 2 min irradiation. Apoptosis characterized by chromatin condensation and cell shrinkage is evident in InCBTN-Cl treated cells (**f**). Inserts present an enlarged view.

Results from cell viability assay shown in Figure 2 indicated that biotin is not toxic to the colon cancer cells in the dark and in the presence of light for concentration of up to 50 μM, which is the maximum concentration used for the assay. Compared to the starting methyl pheophorbide (MePheo) which serves as the control, the chlorin-biotin conjugated derivative (CBTN) increased cell inhibition upon irradiation by approximately 19%. The expected effect was observed, which was that the presence of biotin in the conjugate targeting the vitamin receptors caused a slight increase in cellular toxicity in the colon cancer cell line [15] which is known to have overexpression of biotin receptors. The presence of biotin improved cellular uptake of the photosensitizer causing decreased cell proliferation upon photoirradiation. The indium complex (InCBTN-Cl) compared to either the starting MePheo, the unmetallated CBTN, or the zinc complex ZnCBTN caused a marked decrease in cell proliferation of colon cancer cells by 30% and 40%–50% upon irradiation for 2 and 5 min, respectively. Fluorescence microscopy images of fixed colon cancer cells indicated diffused chromatin and rounded nuclei for unirradiated untreated cells (Figure 3a), and post-treatment with the 5 μM concentration of zinc and indium complexes in the dark in Figure 3c,e, respectively. Upon light irradiation for 2 min of colon carcinoma cells treated with indium complex **5**, apoptotic cells (Figure 3f) are formed as characterized by chromatin condensation and cell shrinkage, and only 40% of the cells survived. Under the same conditions at 2 min, no apoptotic cells (Figure 3b,d) were visible for cells treated with CBTN **3** and the zinc complex **4**. About 70% of the treated cells are still viable after 2 min photosensitization post-treatment with the control MePheo **1**, CBTN **3**, and the zinc complex **4**. Nuclei were stained dark blue with Hoechst 33258. Fluorescence microscopy studies confirmed the results obtained from MTT assay.

Enhanced PDT efficacy of the indium complex could be due to the longer lifetime of excited triplet state generating more cytotoxic singlet oxygen species as observed by others [31,35,37,38,45]. However, the zinc complex ZnCBTN behaved in the same way as the control MePheo, and did not show any improved photodynamic efficacy as the unmetallated chlorin-biotin conjugate CBTN and the indium complex. Insertion of zinc to form the complex ZnCBTN was based on previous studies [30,31,32,33,34] on the treatment of human adenocarcinoma cell lines (A549, MC-7, and LoVo) with the zinc complex of pheophorbide which showed a stronger photodynamic effect than Photofrin (commercially-available photosensitizer) even at low concentration of 5 × 10^−7^ M and light dose of 5 J/cm^2^. This zinc complex required a 20-fold decrease in concentration compared to Photofrin to obtain the desired photodynamic efficiency. Other studies [33] on zinc complex demonstrated efficacy of zinc complexes for PDT which was explained to be due to increased lipophilicity in the presence of zinc in the macrocyclic ring structure resulting in higher vesicle and cell uptake. Enhanced lipophilicity increases PS binding into the mitochondria allowing for favorable membrane interactions which can be ascribed to more efficient membrane photooxidation. Strong membrane interaction of the zinc complex was attributed to a complexing effect with the phosphate groups of the phospholipids particularly in the mitochondrial inner membrane [33]. This increased photodynamic behavior of zinc demonstrated by others upon insertion into the chlorin-biotin conjugate was not observed in this study. In fact, our result indicated that the zinc did not produce any enhancement compared to the unmetallated conjugate. These results suggests that biotin might be forming a complex with zinc ion, and that biotin is not free to target the vitamin receptor that it was intended to.

## 3. Materials and Methods

### 3.1. Chemistry

#### 3.1.1. General

Solvents and reagents were purchased mainly from Sigma Aldrich Chemical Co. (St. Louis, MO, USA). All air and moisture sensitive reactions were performed in anhydrous solvents under nitrogen atmosphere. Chromatographic purifications were performed in normal phase preparative Analtech thin-layer chromatography (TLC) plate (Fisher, Waltham, MA, USA). Reactions were monitored spectrophotometrically or by TLC using polyester backed normal phase analytical plate (Merck, Silica gel 60 F_254_ precoated 200 μm) and detected with UV light (λ = 254 nm). Visible spectra were recorded with an Agilent 8453 UV-visible spectrophotometer. NMR spectra were acquired with a Bruker Avance NMR spectrometer (400 MHz for ^1^H, 100 MHz for ^13^C). Chemical shifts are reported in δ ppm referenced according to the deuterated solvents used as internal standards: CDCl_3_ 7.24 ppm (^1^H), 77.23 ppm (^13^C). High resolution mass spectra were obtained on a Bruker microTOF-II ESI mass spectrometer. All compounds synthesized were isolated and purified in ≥95% purity as confirmed by ^1^H, ^13^C, 2D COSY (Correlated Spectroscopy), DEPT 135 (Distortion-less Enhancement by Polarization Transfer), HSQC (Heteronuclear Single Quantum Correlation) NMR spectra.

#### 3.1.2. Synthesis of 13^1^-Hexamethylenediaminylchlorin e_6_ Dimethyl Ester **2**

Methyl pheophorbide *a* 1 (100 mg, 0.165 mmol) was dissolved in dry CH_2_Cl_2_, and stirred under nitrogen for 10 min. Then *N*-*t*-butyloxycarbonyl-1,6-hexanediamine or *N*-Boc-1,6-hexanediamine (200 mg, 0.93 mmol) dissolved in 1 mL CHCl_3_ (dried over molecular sieves) was added in to the solution and the mixture was stirred for 24 h. The reaction was monitored by TLC (10% acetone/CH_2_Cl_2_) until reaction showed disappearance of the starting methyl pheophorbide *a*. Additional amine may be added to complete the reaction. Solvent was evaporated and the residue was purified by preparative TLC plate using 8% acetone/CH_2_Cl_2_ to afford 130 mg (0.158 mmol, 96% yield) of 13^1^ hexamethylenediaminyl(Boc) chlorin e_6_ DME (C_47_H_62_N_6_O_7_). ^1^H-NMR (CDCl_3_, 400 MHz): δ 9.69 (s, 1H, 10-meso H), 9.64 (s, 1H, 5-meso H), 8.83 (s, 1H, 20-meso H), 8.09–8.02 (dd, *J* = 11.54, 11.80 Hz, 1H, 3^1^C*H*=CH_2_), 6.47 (br s, 1H, –N*H*–CH_2_(CH_2_)_4_CH_2_-NH-), 6.160 (br s, 1H, –NH–CH_2_(CH_2_)_4_CH_2_–N*H*–), 6.35–6.30 (d, *J* = 17.82 Hz, 1H, trans 3^2^CH=C*H*_2_), 6.13–6.10 (d, *J* = 11.54 Hz, 1H, cis 3^2^CH=C*H*_2_), 5.56–5.51 (d, *J* = 18.17 Hz, 1H, 15^1^CH_2_), 5.27–5.23 (d, *J* = 17.08 Hz, 1H, 15^1^CH_2_), 4.57 (br s, 1H, –NH–CH_2_(CH_2_)_4_CH_2_–N*H*–), 4.50–4.45 (q, 1H, 18-H), 4.38–4.35 (m, 1H, 17-H), 4.23–4.21 (br m, 2H, –NH–C*H*_2_(CH_2_)_4_CH_2_–NH–), 3.78 (m, 2H, 8^1^-CH_2_ and s, 3H, 15^1^CO_2_CH_3_), 3.61 (s, 3H, 17^2^CO_2_CH_3_), 3.52 (s, 3H, 12–CH_3_), 3.47 (s, 3H, 2^1^–CH_3_), 3.30 (s, 3H, 7^1^–CH_3_), 3.12 (br m, 2H, –NH–CH_2_(C*H*_2_)_4_C*H*_2_–NH–), 2.56–2.17 (each m, 2H, 17^2^CH_2_, and 2H, 17^1^–CH_2_), 1.78 (br m, 2H, –NH–CH_2_(C*H*_2_)_4_CH_2_–NH–), 1.73–1.68 (m, 3H, 18^1^-CH_3_, and 3H, 8^2^-CH_3_), 1.43 (br s, 9H, Boc-CH_3_), 1.32 (br m, 4H, –NH–CH_2_(C*H*_2_)_4_C*H*_2_–NH–), −1.69 and −1.85 (each br s, 1H each, NH). The 13^1^-hexamethylenediaminyl(Boc) chlorin e_6_ DME (65 mg, 0.085 mmol) was dissolved in dry CH_2_Cl_2_ (4 mL) under nitrogen. Reaction mixture was cooled in an ice bath, and TFA (1.2 mL) was added. Nitrogen was removed and the solution was stirred for 3 h. Complete deprotection was monitored by TLC (10% acetone in CH_2_Cl_2_), and then the solution was washed twice with saturated aqueous NaHCO_3_ solution. After drying over anhydrous Na_2_SO_4_, solvent was removed to yield 50 mg (0.075 mmol, 88% yield) of 13^1^-hexamethylenediaminylchlorin e_6_ DME 2 (C_42_H_54_N_6_O_5_). UV-vis (CH_2_Cl_2_): λ_max_ (ε/M^−^^1^cm^−^^1^) 662 (14493), 622 (1329), 530 (1208), 501 (4227), 402 (46981), 288 (26701); ^1^H NMR (CDCl_3_, 400 MHz): δ 9.41 (s, 1H, 10-meso H), 9.36 (s, 1H, 5-meso H), 8.76 (s, 1H, 20-meso H), 7.72–7.65 (dd, *J* = 17.78, 11.58 Hz, 1H, 3^1^CH=CH_2_), 6.13 (br s, 1H, –N*H*–CH_2_(CH_2_)_4_CH_2_–NH–), 5.99–5.95 (d, *J* = 17.82 Hz, 1H, trans 3^2^CH=CH_2_), 5.71–5.68 (d, *J* = 11.55 Hz, 1H, cis 3^2^CH=CH_2_), 5.29–5.03 (dd, *J* = 18.54, 13.70 Hz, 2H, 15^1^C*H*_2_), 4.47–4.41 (q, 1H, 18-H), 4.28–4.25 (br m, 1H, –NH–CH_2_(CH_2_)_4_CH_2_–N*H*–), 4.23–4.20 (m, 1H, 17–H), 3.60 (s, 3H, 15^1^CO_2_CH_3_), 3.57 (s, 3H, 17^2^CO_2_CH_3_), 3.51–3.46 (br q, 2H, 8^1^-CH_2_), 3.26 (s, 3H, 12^1^-CH_3_), 3.06 (s, 3H, 2^1^-CH_3_), 3.02 (s, 3H, 7^1^-CH_3_), 2.76 (br m, 4H, –NH–C*H*_2_(CH_2_)_4_C*H*_2_NH–), 2.59–2.17 (each m, 2H, 17^2^CH_2_, and 1H, 17^1^-CH_2_), 1.76–1.72 (br m, 8H, –NH–CH_2_(C*H*_2_)_4_CH_2_NH–), 1.67–1.65 (d, 3H, 18^1^-CH_3_), 1.60–1.57 (t, 3H, 8^2^-CH_3_), 1.44–1.32 (br m, 6H, –NH–CH_2_(C*H*_2_)_4_CH_2_NH–), −1.74 and −2.00 (each br s, 1H each, NH); ^13^C-NMR (CDCl_3_, 100 MHz): δ 174.2 (17^2^-*C*O_2_CH_3_), 173.5 (15^1^-*C*O_2_CH_3_), 169.0 (13-*C*ONH), 167.8 (19), 166.8 (1), 162.3 (4), 154.0 (6), 148.9 (14), 144.6 (16), 145.4 (3), 143.5 (8), 138.7 (9), 136.1 (11), 134.9 (2), 134.7 (12), 134.3 (7), 130.9 (3^1^), 129.7 (15), 128.8 (10), 128.0 (13), 121.4 (3^2^), 101.2 (5), 98.8 (20), 53.1 (17), 52.3 (15^1^CO_2_*C*H_3_), 51.6 (17^2^CO_2_*C*H_3_), 49.2 (18), 39.5 (–NH–*C*H_2_(CH_2_)_4_CH_2_–NH–), 39.0 (–NH–CH_2_(CH_2_)_4_*C*H_2_–NH–), 37.5 (15^1^), 29.4 (17^2^), 29.1 (17^1^), 28.8 (–NH–CH_2_(*C*H_2_)_4_CH_2_–NH–), 26.9 (–NH–CH_2_(*C*H_2_)_4_CH_2_–NH–), 25.6–24.6 (–NH–CH_2_(*C*H_2_)_4_CH_2_NH–), 19.9 (18^1^), 19.5 (8^1^), 17.6 (8^2^), 14.1 (12^1^), 13.8 (2^1^), 13.1 (7^1^); HRMS (MALDI-TOF) *m*/*z* 723.4214 [M]^+^, calcd for C_42_H_54_N_6_O_5_ 723.4228.

#### 3.1.3. Synthesis of 13^1^-Hexamethylenediaminyl-Biotinylchlorin e_6_ Dimethyl Ester, CBTN, **3**

In a dry round bottom flask containing 13^1^-hexamethylenediaminylchlorin e_6_ DME (92 mg, 0.128 mmol), biotin (48 mg, 0.138 mmol), 4-(4,6-dimethoxy-1,3,5-triazin-2-yl)-4-methylmorpholinium chloride (DMTMM, 42 mg, 0.150 mmol) was stirred in dry CH_2_Cl_2_ (25 mL) under nitrogen overnight for 12 h. The reaction was monitored by TLC (10% methanol in CH_2_Cl_2_) until reaction showed disappearance of the starting amine. Solvent was evaporated and the residue was purified by preparative TLC plate using the same solvent system to afford 76 mg (0.072 mmol, 57% yield) of 13^1^-hexamethylenediaminyl-biotinylchlorin e_6_ DME, CBTN, 3 (C_52_H_68_N_8_O_7_S). UV-vis (CH_2_Cl_2_): λ_max_ (ε/M^−^^1^cm^−^^1^) 663 (14509), 607 (1373), 527 (1292), 502 (4290), 402 (47174), 289 (27004); ^1^H-NMR (CDCl_3_, 400 MHz): δ 9.69 (s, 1H, 10-meso H), 9.61 (s, 1H, 5-meso H), 8.82 (s, 1H, 20-meso H), 8.13–8.05 (dd, *J* = 17.75, 11.58 Hz, 1H, 3^1^C*H*=CH_2_), 7.32 (br s, 1H, -N*H*CH_2_(CH_2_)_4_CH_2_NH-), 6.37–6.30 (d, *J* = 17.82 Hz, 1H, trans 3^2^CH=C*H*_2_), 6.18–6.13 (d, *J* = 11.55 Hz, 1H, cis 3^2^CH=C*H*_2_), 5.98 (br m, 1H, –NH–CH_2_(CH_2_)_4_CH_2_N*H*–), 5.58–5.38 (dd, *J* = 18.54, 13.70 Hz, 2H, 15^1^CH_2_), 5.15 (br m, 2H_a-b_, –C*H*NHCONHC*H*– in biotin ring), 4.71 (br m, 2H, –CHN*H*CON*H*CH– in biotin ring), 4.45 (q, 1H, 18-H), 4.39 (m, 1H, 17-H), 3.79 (s, 3H, 15^1^CO_2_CH_3_), 3.78 (br q, 2H, 8^1^-CH_2_), 3.67 (s, 3H, 17^2^CO_2_CH_3_), 3.58 (br t, 2H, –NHC*H*_2_(CH_2_)_4_CH_2_NH–), 3.56 (s, 3H, 12^1^-CH_3_), 3.51 (s, 3H, 2^1^-CH_3_), 3.43 (br t, 2H, –NHCH_2_(CH_2_)_4_C*H*_2_NH–), 3.25 (br m, 1H_d_, –CH_2_C*H*–S– in biotin ring; and, 3H, 7^1^-CH_3_), 3.13–2.93 (br dd, 2H_a_, –SC*H*_2_– in biotin ring), 2.20–2.15 (br m, 2H, 17^2^C*H*_2_; 2H, 17^1^-CH_2_; and 2H, –NHCOC*H*_2_(CH_2_)_3_–biotin), 1.75–1.73 (br m, 2H, –NHCH_2_(C*H*_2_)_4_CH_2_NH–), 1.67–1.65 (d, 3H, 18^1^-CH_3_), 1.63–1.60 (t, 3H, 8^2^-CH_3_), 1.50–1.32 (br m, 6H, –NHCH_2_(C*H*_2_)_4_CH_2_NH–; and 6H, –NHCOCH_2_(C*H*_2_)_3_–biotin), −1.82 and −2.01 (each br s, 1H each, NH); ^13^C-NMR (CDCl_3_, 100 MHz): δ 175.8 (C=O biotin ring), 173.6 (17^2^-*C*O_2_CH_3_ and 15^1^-*C*O_2_CH_3_), 173.2 (13-*C*ONH), 170.6 (hexyl-NH*C*O), 164.5 (19), 161.6 (1), 160.0 (4), 152.2 (6), 147.2 (14), 146.2 (16), 145.4 (3), 143.5 (8), 141.4 (9), 140.9 (11), 137.8 (2), 133.6 (12), 130.9 (7), 126.7 (3^1^), 124.5 (15), 119.7 (3^2^), 102.5 (10), 101.3 (13), 100.5 (5), 96.6 (20), 58.1 (*C*_b_H, biotin ring), 57.9 (*C*_c_H, biotin ring), 55.9 (*C*_d_H, biotin ring, next to S), 54.1 (17), 53.5 (15^1^CO_2_*C*H_3_), 51.5 (17^2^CO_2_*C*H_3_), 45.1 (18), 42.6 (*C*_a_H_2_, biotin ring, next to S), 42.5 (–NH–*C*H_2_(CH_2_)_4_CH_2_–NH–), 40.5 (–NH–CH_2_(CH_2_)_4_*C*H_2_–NH–), 39.8 (–NH–(CH_2_)_6_–NHCO–*C*H_2_–), 37.9 (–NH–(CH_2_)_6_–NHCO–(CH_2_)_2_*C*H_2_–), 37.1 (15^1^), 29.4 (17^2^), 29.1 (17^1^), 28.8 (–NH–CH_2_(*C*H_2_)_4_CH_2_–NH–), 28.2 (–NH–(CH_2_)_6_–NHCO–CH_2_(*C*H_2_)CH_2_–), 26.9 (–NH–CH_2_(*C*H_2_)_4_CH_2_–NH–), 26.4 (–NH–(CH_2_)_6_–NHCO–(CH_2_)_3_*C*H_2_–), 25.6–24.6 (–NH–CH_2_(*C*H_2_)_4_CH_2_NH–), 19.9 (18^1^), 19.5 (8^1^), 17.6 (8^2^), 14.1 (12^1^), 13.8 (2^1^), 13.1 (7^1^); HRMS (MALDI-TOF) *m*/*z* 949.6150 [M^+^], calcd for C_52_H_68_N_8_O_7_S 949.2311.

#### 3.1.4. Synthesis of Zn(II)-13^1^-Hexamethylenediaminyl-Biotinylchlorin e_6_ Dimethyl Ester, ZnCBTN, **4**

In a dry round bottom flask, CBTN 3 (50 mg, 0.053 mmol) was dissolved in 5 mL methanol. Saturated methanolic solution of zinc(II) acetate (5 mL) were added to the mixture and stirred for 2 h. After checking spectrophotometrically for completion, the reaction mixture was washed with saturated aqueous solution of sodium bicarbonate, followed by washing with water (3 × 50 mL), and extracted with dichloromethane. The organic layer was dried over anhydrous sodium sulfate and the organic solvent was evaporated under high vacuum. The crude product was purified by preparative TLC plate using 3% methanol-dichloromethane to afford 40 mg (0.039 mmol, 73% yield) of the title compound 4. UV-vis (CH_2_Cl_2_): λ_max_ (ε/M^−^^1^cm^−^^1^) 637 (57392), 590 (12101), 414 (145914); ^1^H NMR (CDCl_3_, 400 MHz): δ 9.49 (s, 1H, 10-meso H), 9.46 (s, 1H, 5-meso H), 8.50 (s, 1H, 20-meso H), 8.07–7.99 (dd, *J* = 17.81, 11.55 Hz, 1H, 3^1^C*H*=CH_2_), 6.93 (br s, 1H, –N*H*CH_2_(CH_2_)_4_CH_2_NH–), 6.45 (br t, 1H, –NHCH_2_(CH_2_)_4_CH_2_N*H*–), 6.18–6.14 (d, *J* = 7.55 and 1.14 Hz, 1H, trans 3^2^CH=C*H*_2_), 5.99–5.96 (d, *J* = 10.89 and 0.87 Hz, 1H, cis 3^2^CH=C*H*_2_), 5.45–4.92 (dd, *J* = 18.19, 13.70 Hz, 2H, 15^1^C*H*_2_), 4.32 (q, 1H, 18-H), 4.22 (br m, 2H, –N*H*–CON*H*– in biotin ring), 4.18 (m, 1H, 17-H), 3.83 (s, 3H, 15^1^CO_2_C*H*_3_), 3.79 (br m, 2H_b-c_, –C*H*NHCONHC*H*– in biotin ring, and 2H, 8^1^-CH_2_), 3.76–3.62 (br m, 2H, –CHN*H*CON*H*CH– in biotin ring), 3.50 (s, 3H, 17^2^CO_2_CH_3_), 3.34 (s, 3H, 12^1^-CH_3_; and, 3H, 7^1^-CH_3_), 3.32 (s, 3H, 2^1^-CH_3_), 3.30 (br m, 4H, –NHC*H*_2_(CH_2_)_4_C*H*_2_NH– and, 1H_d_, –CH_2_C*H*–S– in biotin ring), 3.06–2.96 (br dd, 2H_a_, –SC*H*_2_– in biotin ring), 2.26 (br m, 2H, 17^2^C*H*_2_), 2.13–2.04 (two m, 2H, 17^1^-CH_2_; and 2H, –NHCOC*H*_2_(CH_2_)_3_–biotin), 1.95–1.86 (br m, 2H, –NH–CH_2_(C*H*_2_)_4_CH_2_NH–), 1.73–1.70 (d, 3H, 18^1^-CH_3_), 1.69–1.65 (t, 3H, 8^2^-CH_3_), 1.43–1.24 (br m, 6H, –NH–CH_2_(C*H*_2_)_4_CH_2_NH–; and 6H, –NHCOCH_2_(C*H*_2_)_3_–biotin); ^13^C-NMR (CDCl_3_, 100 MHz): δ 174.7 (C=O biotin ring), 173.9 (17^2^-*C*O_2_CH_3_ and 15^1^-*C*O_2_CH_3_), 173.7 (13-*C*ONH), 171.8 (hexyl-NH*C*O), 165.0 (19), 161.3 (1), 160.1 (4), 152.9 (6), 147.9 (14), 146.9 (16), 145.5 (3), 143.7 (8), 141.7 (9), 140.9 (11), 138.5 (2), 133.6 (12), 133.3 (7), 130.6 (3^1^), 128.8 (15), 119.5 (3^2^), 102.5 (10), 101.3 (13), 100.5 (5), 93.1 (20), 68.2 (*C*_b_H, biotin ring), 60.9 (*C*_c_H, biotin ring), 58.3 (*C*_d_H, biotin ring, next to S), 54.5 (17), 52.1 (15^1^CO_2_*C*H_3_), 51.5 (17^2^CO_2_*C*H_3_), 47.5 (18), 38.9 (*C*_a_H_2_, biotin ring, next to S), 38.3 (–NH–*C*H_2_(CH_2_)_4_CH_2_–NH–), 38.1 (–NH–CH_2_(CH_2_)_4_*C*H_2_–NH–), 37.7 (–NH–(CH_2_)_6_–NHCO–*C*H_2_–), 36.3 (15^1^), 30.4 (17^2^), 29.5 (17^1^), 29.3 (–NH–(CH_2_)_6_–NHCO–(CH_2_)_2_*C*H_2_–), 28.6 (–NH–(CH_2_)_6_–NHCO–CH_2_(*C*H_2_)CH_2_–), 28.2 (–NH–CH_2_(*C*H_2_)_4_CH_2_–NH–), 26.4 (–NH–(CH_2_)_6_–NHCO–(CH_2_)_3_*C*H_2_–), 25.6–24.6 (–NH–CH_2_(*C*H_2_)_4_CH_2_–NH–), 22.8 (18^1^), 19.5 (8^1^), 17.8 (8^2^), 12.3 (12^1^), 12.1 (2^1^), 11.2 (7^1^); HRMS (MALDI-TOF) *m*/*z* 1033.3986 [M^+^ + Na], calcd for C_52_H_66_N_8_NaO_7_SZn 1033.3959.

#### 3.1.5. Synthesis of 13^1^-Hexamethylenediaminyl-Biotinylchlorin e_6_ Dimethyl Ester Indium (III) Chloride, InCBTN-Cl, **5**

In a dry round bottom flask, CBTN 3 (50 mg, 0.053 mmol) was dissolved in 15 mL toluene, then sodium acetate (500 mg), anhydrous potassium carbonate (500 mg) and indium chloride (300 mg) were added. The reaction mixture was refluxed under nitrogen atmosphere overnight. After checking spectrophotometrically for complete metal insertion, the reaction mixture was neutralized with acetic acid and washed with water (3 × 50 mL), then the organic layer was dried over anhydrous sodium sulfate. Solvent was evaporated under high vacuum. The crude product was purified by preparative TLC plate using 3% methanol-dichloromethane to afford 45 mg (0.041 mmol, 77% yield) of the title compound 5. UV-vis (CH_2_Cl_2_): λ_max_ (ε/M^−^^1^cm^−^^1^) 638 (52876), 581 (7032), 413 (165205); ^1^H NMR (CDCl_3_, 400 MHz): δ 9.61 (br s, 2H, 10-meso H, and 5-meso H), 8.61 (s, 1H, 20-meso H), 7.98–7.89 (dd, *J* = 17.81, 11.55 Hz, 1H, 3^1^C*H*=CH_2_), 6.20–6.15 (d, *J* = 7.55 and 1.14 Hz, 1H, trans 3^2^CH=C*H*_2_), 6.07–6.03 (d, *J* = 10.89 and 0.87 Hz, 1H, cis 3^2^CH=C*H*_2_), 5.21 (br m, 2H, 15^1^CH_2_), 4.39 (q, 1H, 18-H), 4.29 (br m, 2H, –N*H*–CON*H*– in biotin ring), 4.16 (m, 1H, 17-H), 3.71 (s, 3H, 15^1^CO_2_C*H*_3_), 3.70 (br m, 2H_b-c_, –C*H*NHCONHC*H*– in biotin ring, and 2H, 8^1^-C*H*_2_; and, 2H, -CHN*H*CON*H*CH- in biotin ring), 3.33 (br s, 3H, 17^2^CO_2_CH_3_; 3H, 12^1^-CH_3_; and, 3H, 7^1^-C*H*_3_), 3.27 (s, 3H, 2^1^-CH_3_), 3.19 (br m, 4H, –NHC*H*_2_(CH_2_)_4_C*H*_2_NH–; 1H_d_, –CH_2_C*H*–S– in biotin ring; and, 2H_a_, –SC*H*_2_– in biotin ring), 2.23 (br m, 2H, 17^2^CH_2_), 2.16–2.11 (two m, 2H, 17^1^-CH_2_; and 2H, –NHCOC*H*_2_(CH_2_)_3_–biotin), 1.95–1.74 (br m, 2H, –NH–CH_2_(C*H*_2_)_4_CH_2_NH–), 1.63–1.60 (m, 3H, 18^1^-CH_3_; and 3H, 8^2^-CH_3_), 1.51–1.34 (br m, 6H, –NH–CH_2_(C*H*_2_)_4_CH_2_NH–; and 6H, –NHCOCH_2_(C*H*_2_)_3_–biotin); ^13^C-NMR (CDCl_3_, 100 MHz): δ 174.2 (C=O biotin ring), 173.9 (17^2^-*C*O_2_CH_3_ and 15^1^-*C*O_2_CH_3_), 173.5 (13-*C*ONH), 173.4 (hexyl-NH*C*O), 167.8 (19), 161.3 (1), 159.1 (4), 153.9 (6), 148.1 (14), 146.9 (16), 145.6 (3), 143.7 (8), 142.9 (9), 140.7 (11), 139.5 (2), 133.1 (12), 132.5 (7), 130.7 (3^1^), 128.8 (15), 119.5 (3^2^), 102.8 (10), 101.1 (13), 100.2 (5), 93.6 (20), 68.2 (*C*_b_H, biotin ring), 60.7 (*C*_c_H, biotin ring), 58.3 (*C*_d_H, biotin ring, next to S), 54.5 (17), 52.4 (15^1^CO_2_*C*H_3_), 51.8 (17^2^CO_2_*C*H_3_), 40.3 (18), 38.8 (*C*_a_H_2_, biotin ring, next to S), 38.5 (–NH–*C*H_2_(CH_2_)_4_CH_2_–NH–), 38.2 (–NH–CH_2_(CH_2_)_4_*C*H_2_–NH–), 37.7 (–NH–(CH_2_)_6_–NHCO–*C*H_2_–), 36.1 (15^1^), 30.1 (17^2^), 29.8 (17^1^), 29.1 (–NH–CH_2_(*C*H_2_)_4_CH_2_–NH–), 28.6 (–NH–(CH_2_)_6_–NHCO–CH_2_(*C*H_2_)_2_–), 28.1 (–NH–CH_2_(*C*H_2_)_4_CH_2_–NH–), 26.4 (–NH–(CH_2_)_6_–NHCO–(CH_2_)_3_*C*H_2_–), 26.4–25.1 (–NH–CH_2_(*C*H_2_)_4_CH_2_–NH–), 23.0 (18^1^), 19.5 (8^1^), 17.5 (8^2^), 14.1 (12^1^), 12.2 (2^1^), 10.9 (7^1^); HRMS (MALDI-TOF) *m*/*z* 1061.3804 [M^+^ − Cl], calcd for C_52_H_66_ClInN_8_O_7_S 1097.3575.

### 3.2. In Vitro Cytotoxicity Assay

#### 3.2.1. General

Mouse colon carcinoma cell line CT26. WT was purchased from the American Type Culture Collection (ATCC CRL-2638). CT26 cells were culutred in RPMI 1640 medium (ATCC) and were grown to 80%–90% confluence in 75-cm^2^ culture flasks (Corning, Corning, NY, USA) for about a week (5–6 days) in a humidified incubator (Fisher Scientific Isotemp, Waltham, MA, USA) with 5% CO_2_ at 37 °C. During the incubation period, growth media was changed once with fresh pre-warmed medium (pH 7.2). To harvest the cells, old growth media was aspirated and 5 mL 0.25% trypsin solution (Thermo Sci Hyclone, Waltham, MA, USA) were added. The cells were incubated for 10 min and gently scraped to detach cells from the flask walls. Clumped cells were broken up gently and 1 mL of the trypsinized cell homogenate suspension was transferred into a new T75 cell culture flask containing pre-warmed media (20 mL) for further culturing.

#### 3.2.2. Cell Survival Assay

Cells were grown to confluence in a 96-well plate (4 × 10^4^ cells/well) and treated for 24 h with compounds or photosensitizers (0.5, 1, 5, 10, 25, 50 μM) in growth media from a stock solution of 10 mM in DMSO (dimethyl sulfoxide, Fisher, Waltham, MA USA). After 24 h treatment, cells were washed with fresh media and positioned below a non-coherent LumaCare LC-122 650 nm light source for 1, 2, and 5 min at an energy fluence rate of 16 mW/cm^2^ (measured using a Newport optical power meter Model 840). Unirradiated cells served as control samples. The following day, cells were washed with pre-warmed PBS, and MTT (3-[4,5-dimethyl-thiazol-2-yl]-2.5-diphenyltetrazolium bromide, Sigma, St. Louis, MO, USA, 0.3 mg/mL) in PBS was added to each well. Samples were allowed to incubate for additional 2 h, after which dark blue crystals formed. DMSO was added to each well and allowed to stand at room temperature for 1 h to dissolve the purplish-blue formazan crystals. Plates were shaken for 2 min and thereafter the absorbance values at 490 nm were measured on a microplate reader (BioRad 550, Hercules, CA, USA). Absorbance readings were calculated based on the absorbance of the unirradiated cells alone (as control) and were directly proportional to the number of viable cells in the culture. Results were reported as the average of triplicate measurements.

#### 3.2.3. Fluorescence Microscopy

Cells (1 mL aliquots) obtained from a diluted cell suspension were seeded into each well (1.7 cm^2^, ~1000 cells/well) of a 4-well culture slide (BD Biosciences, San Jose, CA, USA) and grown to confluence in 5% CO_2_ at 37 °C for 6–7 days for attachment to the substratum. After aspirating the old growth media, 1 mL of the compound or photosensitizer (5 μM) in fresh pre-warmed media at 37 °C was added to each well. After photosensitizer treatment for 24 h, cells were washed twice with 1 mL fresh growth media, and then irradiated with light using LumaCare LC-122 as described above. Cells were stained in the dark with 1 mL of 0.1 mg/mL Hoechst 33258 (Molecular Probes) in pre-warmed media for 10 min at 37 °C, washed twice with 1 mL filtered PBS, then fixed with 1 mL filtered pre-warmed 4% paraformaldehyde for 15 min in the incubator. After thorough liquid aspiration, the wells were removed and allowed to air dry in the dark for 1 h. Slides were protected with coverslips, whose edges were sealed using a clear fast-drying nail polish and allowed to dry at room temperature in the dark for 30 min. Images were recorded by means of fluorescence microscopy (DAPI for Hoechst 350–390 nm excitation and 460–490 nm emission filters) using an upright fluorescence microscope with Retiga imaging 2000R (Nikon Optiphot-2, 20× and 40×, Nikon, Chiyoda, Japan) and an image processing Nikon NIS-Elements V4.0 Qimaging software (Nikon, Chiyoda, Japan).

## 4. Conclusions

Vitamin receptors over-expressed in cancer cells are potential targets for effective delivery of anti-cancer drugs. Results in our study indicated that biotin provided a means for increased uptake of photosensitizers to cause decreased cancer cell survival. Chelating the biotin-conjugated photosensitizer forming the indium complex provided additional PDT efficacy in vitro by about 30% compared to the unmetallated biotin-chlorin complex. However, the zinc complex of the biotin-chlorin conjugate did not significantly improve photodynamic efficacy in colon cancer cell line compared to the unmetallated complex. Further work is required to determine if the biotin-conjugated photosensitizers synthesized in this study will improve internalization of the photosensitizers in other cancer cell lines that exhibit over-expression of biotin receptors including ovarian, breast, renal, lung, and leukemia cell lines. Future studies can include determination of singlet oxygen (^1^O_2_) quantum yield of the synthesized chlorin-biotin conjugates.
